# Regional disparities in the distribution of public and private healthcare facilities in South Korea

**DOI:** 10.1371/journal.pone.0330090

**Published:** 2025-09-24

**Authors:** Jiyu Park, Byeongyun Jeon, Eun Lee

**Affiliations:** Department of Scientific Computing, Pukyong National University, Busan, Republic of Korea; Chunghwa Telecom Co. Ltd., TAIWAN

## Abstract

Unequal healthcare facility distributions remain a critical concern in South Korea, where medical resources are disproportionately concentrated in metropolitan areas, leading to disparities in the availability of services between regions. These spatial disparities are also influenced by broader contextual factors such as socioeconomic inequality and transportation infrastructure, which interact with access to care. Focusing on the spacial dimension of these disparities, this study investigates the spatial distribution of public and private medical facilities by analyzing their allocation patterns to identify underlying principles associated with these distributional trends, using a cost optimization approach rooted in statistical physics. By employing concentration index (CI), we quantify regional inequalities in general hospitals and clinics. Additionally, using scaling exponents, we aim to understand the principles underlying the distributional differences between public and private healthcare facilities and examine whether these differences vary across medical specialties. Our findings reveal that private hospitals and clinics exhibit stronger correlations with population density, reflecting commercial-like allocation patterns, while public institutions demonstrate more even spatial distributions. However, some essential medical specialties, including emergency medicine, pediatrics, and obstetrics and gynecology, are sparsely distributed in public sectors. Furthermore, temporal scaling exponent analysis suggests that private medical institutions are gradually intensifying their commercial characteristics, potentially exacerbating future disparities. These results provide a comprehensive understanding of the distribution patterns of healthcare facilities across regions and establishment types, while uncovering the underlying principles that shape these patterns based on medical specialties, providing valuable insights to inform future resource allocation strategies in the health sector.

## Introduction

The unequal distribution of healthcare facilities remains a global issue, with persistent disparities not only between urban and rural areas but also across socioeconomic groups and regions with inadequate infrastructure [1–4]. These global patterns are reflected in South Korea, where the regional imbalance is particularly pronounced. Major healthcare resources remain concentrated in metropolitan areas, leaving many non-metropolitan regions underserved [5]. These disparities in healthcare facilities can not only hinder access to essential medical services but also contribute to differences in health outcomes. For example, the uneven spatial distribution of the facilities limits patient choices, with a quarter of patients traveling outside their immediate district for treatment [6]. Moreover, these distributional gaps are linked to higher mortality rates in non-metropolitan regions compared to metropolitan areas [7]. Therefore, reducing regional disparities in the distribution of healthcare institutions can improve health equity for all. To achieve this, it is crucial to examine how healthcare resources are distributed and, more importantly, understand why these patterns emerge [8–11].

One key factor influencing these distribution patterns is the characteristics of healthcare institutions, including their establishment purpose and medical specialization. Based on their establishment type, they can be broadly categorized as either public or private. Public hospitals are generally intended to provide broad and equitable access to essential healthcare services, whereas private hospitals tend to operate on a profit-driven basis, leading to their concentration in economically viable regions.

In addition, medical specialization is also correlated with distinct regional distribution patterns. For example, the utilization of cardiovascular surgery is lower in rural regions than in urban areas, leading to worse health outcomes related to cardiovascular disease [12]. Similarly, maternal mortality rates remain disproportionately high in areas with limited access to obstetric care [13]. Specialized services—such as cardiovascular surgery or obstetrics—often require advanced infrastructure (e.g., specialized operating theaters, high-tech diagnostic equipment) and sufficient patient volume to be economically viable, causing them to cluster in urban centers [14,15]. However, despite these disparities and the importance of understanding specialty-level distribution, there is a lack of research on how the distribution of specialties varies by medical specialty and establishment type, particularly in South Korea. This knowledge gap limits our understanding of the underlying factors driving regional disparities in healthcare access, making it difficult to develop effective policies for equitable healthcare distribution.

To systematically examine the relationship between institutional characteristics and regional distribution disparities, this study quantifies the distribution of public and private healthcare facilities across administrative regions while considering medical specialties. By utilizing the spatial concentration index (CI), which captures how unequally resources are distributed across regions, we quantify disparities in the allocation of medical institutions and assess the extent to which these patterns align with population distribution. In addition, by analyzing hospital distribution at different administrative scales (provincial vs. municipal levels), we identify how regional segmentation affects inequality metrics.

Beyond measuring inequality, this study seeks to uncover the underlying principles shaping the distribution patterns of healthcare facilities. In addition to spatial disparities, we also examine how distributional patterns have evolved over time through a temporal scaling analysis, offering insights into ongoing structural shifts in both public and private healthcare sectors. To achieve this, we employ a scaling analysis approach—commonly used in complex systems—to examine how sensitively healthcare facility density responds to population density, capturing the elasticity of this spatial relationship [16].

Specifically, we analyze the relationship between population density and density of healthcare facilities (e.g., general hospitals and clinics) using scaling exponents (*α*) to characterize how different types of medical institutions respond to population concentration. This approach reveals distinct allocation patterns, ranging from “public-oriented” to “commercial-like” distributions, providing insights into their underlying principles. Finally, this study examines temporal changes in distribution patterns of general hospitals and clinics by analyzing variations in scaling exponents over time, providing insights into the evolving trends of public and private healthcare institutions across medical specialties.

These results will offer a systematic framework for understanding regional disparities in the distribution patterns of healthcare facilities, providing insights for improving healthcare accessibility policies and addressing regional disparities in medical services in South Korea and other countries with similar healthcare environments.

## Materials and methods

To examine the distribution patterns of healthcare facilities in South Korea, we classified them into general hospitals and clinics, as detailed in the following paragraphs, and collected distribution data from government sources. In addition to describing the data, we specify the preprocessing procedures for population and administrative data, along with detailed descriptions of the inequality measures applied in this study.

### Categories of healthcare institutions

#### Distinction between general hospitals and clinics.

We narrowed the scope of healthcare institutions to medical institutions and used the classification defined by South Korea’s Medical Service Act. In addition, please note that we use “healthcare facilities” and “medical institutions” interchangeably in this study to refer to the two main categories of medical institutions: general hospitals and clinics.

The legal categories in the Medical Service Act in South Korea are as follows: (1) Tertiary Hospitals, which have more than 500 beds and provide specialized medical services across various departments, including internal medicine, surgery, obstetrics and gynecology, pediatrics, and emergency care; (2) General Hospitals, defined as hospitals with at least 100 but fewer than 500 beds, offering a broad range of medical services with fewer specialized departments compared to tertiary hospitals; (3) Community Hospitals, which have at least 30 but fewer than 100 beds, providing essential medical services across multiple specialties; and (4) Clinics, small medical institutions with fewer than 30 beds, typically operated by individual practitioners and offering basic healthcare services in limited specialties, without inpatient or emergency care.

In this study, “general hospitals” specifically refer to hospitals in category (2), excluding tertiary hospitals in category (1) as shown in Table 1. Tertiary hospitals are excluded from the analysis due to their substantially larger scale—characterized by a higher number of beds, physicians, medical services, and specialized departments—which differentiates them from local general hospitals and results in nationwide patient inflow, making direct comparison inappropriate. Additionally, “community hospitals” in category (3) and “clinics” in category (4) are collectively referred to as “clinics,” and the analysis was conducted by comparing these clinics with general hospitals. This grouping is based on the fact that community hospitals and clinics are similar in operational scale and patient accessibility characteristics, distinguishing them collectively from larger general hospitals. For the detailed number institutions for each type, please refer to Table 1.

**Table 1:** National distribution of medical institutions by type. The data represent the nationwide distribution of various types of medical institutions based on 2024 statistics [17]. The first column, Medical Institution Type, categorizes the types of medical institutions, while the second and third columns, Private and Public, present the number of private and public hospitals, respectively. The fourth column, Classification in this Study, indicates how each dataset is used in the analysis. In this study, ‘Tertiary Hospitals’ are excluded, and ‘Community Hospitals, and Clinics’ are collectively referred to as ‘clinics’.

Medical institution type	Private	Public	Classification in this study
Tertiary Hospitals	5	12	-
General Hospitals	233	61	General hospital
Community Hospitals	1,358	26	Clinics
Clinics	36,126	110	

#### Distinction between public and private healthcare institutions.

To test whether establishment types (e.g., public and private) are correlated with distributional differences in medical institutions, we classified them as either public or private (Table 1). Private institutions are established by individuals, incorporated associations, social welfare corporations, social cooperatives, consumer cooperatives, medical corporations, foundations, religious organizations, and corporate entities. In contrast, public institutions include national, public, public-national, special corporations, and municipal (city/county) and provincial entities.

Although we classify healthcare institutions as either public or private in this study, we acknowledge that there are meaningful variations within these categories—for example, distinctions between national and municipal public hospitals or between for-profit and non-profit private hospitals. Due to the structure of the available data and the scope of our analysis, we do not differentiate among these subtypes here, but we consider this an important area for future research.

#### Medical specialties.

We defined medical specialties based on the governmental classification as follows: Internal Medicine (IM), Surgery, Emergency Medicine (EM), Thoracic Surgery (CTS), Urology, Pediatrics (Peds.), Obstetrics and Gynecology (OB/GYN), Psychiatry (Psych.), Dermatology (Derm.), and Plastic Surgery (Plast. Surg.). Since clinics do not have emergency medicine departments, the clinic dataset does not include data on emergency medicine. In the analysis of medical specialties, a single healthcare institution may appear under multiple specialties if it offers services across several specialty areas.

### Data sources

#### Administrative regions.

Among the administrative classifications in South Korea, which range from smaller to larger areas—eup, myeon, and dong (sub-municipal level), si, gun, and gu (municipal level), and do and metropolitan cities (provincial level)—this study focuses on the si, gun, gu, and do levels, corresponding to the municipal and provincial levels.

To analyze medical institution distribution patterns, regions were grouped into metropolitan and non-metropolitan areas in accordance with the Local Autonomy Act and its enforcement regulations, which classify metropolitan cities—including special self-governing cities such as Sejong—as urban administrative divisions with populations over 500,000 and administrative autonomy [18,19]. The metropolitan regions include Seoul, Busan, Incheon, Daegu, Daejeon, Gwangju, and Ulsan, as well as Sejong City and Jeju Special Self-Governing Province, which are also classified as metropolitan areas (S1 Fig). The other regions—Gyeonggi-do, Gangwon-do, Chungcheongbuk-do, Chungcheongnam-do, Jeollabuk-do, Jeollanam-do, Gyeongsangbuk-do, and Gyeongsangnam-do—are classified as non-metropolitan administrative areas, as shown in S1 Fig. For consistency and simplicity, regional names were abbreviated as follows: South Korea (SK), Seoul Metropolitan City (Seoul), Jeju Special Self-Governing Province (Jeju), and Sejong City (Sejong).

#### Healthcare data sources and key information.

The data on the number of healthcare institutions by year were obtained from the Public Data Portal, a government-managed data repository in South Korea [17]. For the temporal analysis of the institution’s distribution, we collected healthcare data from December 2021, March, June, October, and December 2022, December 2023, and March and June 2024. The dataset used for analysis includes details such as the type of healthcare institution, administrative divisions, geographic coordinates, medical specialties, and the institution’s founding entity. Based on this information, we classified institutions as either public or private, as described in the Methods section.

#### Population and area data sources.

Population data by administrative regions (provincial and municipal levels) were collected from the Resident Registration Population Statistics Service, provided by the Ministry of the Interior and Safety of South Korea [20]. Under the current administrative district definitions, all regions had complete records, and the analysis utilized the full set of population and affiliated healthcare facility data nationwide.

### Key analytical measures

#### Defining voronoi cells and measuring their areas.

**Fig 1:**
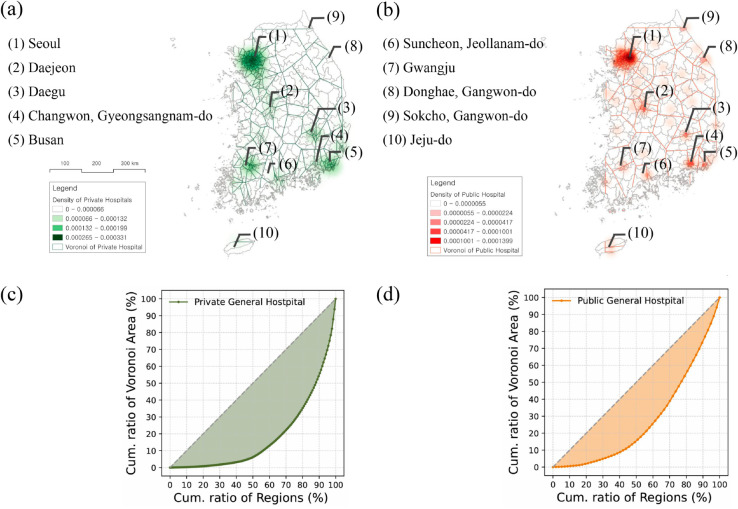
Spatial distribution of general hospitals visualized through Voronoi cells. Voronoi maps illustrate the distribution of private general hospital facilities (a) and public general hospital facilities (b) across 250 municipal areas in South Korea in 2024. Darker colors in the Voronoi regions indicate a higher density of general hospitals, and a larger Voronoi cell area signifies that a single general hospital serves a wider region. The top 10 most highly concentrated regions are labeled with numbers based on the distribution of public hospitals in panel (b). Panels (c) and (d) display the Lorenz curves for the Voronoi areas of private and public general hospitals, respectively. The *x*-axis represents the cumulative percentage of regions, sorted in ascending order by the number of facilities, while the *y*-axis represents the cumulative percentage of the Voronoi area. The area under the diagonal equity line corresponds to the Gini coefficient, which is 0.64 for private general hospitals and 0.45 for public general hospitals, respectively.

With the precise geographical coordinates of each healthcare institution *i*, a Voronoi cell $V_{i}$ can be generated, encompassing all locations that are closer to *i* than to any other facility [21]. In this study, we applied the geographic locations of medical institutions to QGIS (Quantum Geographic Information System) and used the GEOS (Geometry Engine - Open Source) library to calculate the Voronoi cells. This process utilizes the Delaunay triangulation algorithm [22], which employs bisectors between pairs of points to define the boundaries of Voronoi cells. As a result, each Voronoi cell $V_{i}$ contains a single medical institution and represents the coverage area, as shown in Fig 1(a) and 1(b). To analyze disparities in the spatial coverage of the institutions, the area of each Voronoi cell was calculated using the coordinates of its polygon vertices on the Cartesian plane, as shown in Eq 1.

$$A_{i}=\frac{1}{2}\left|\sum_{k=1}^{K}(x_{k}y_{k+1}-x_{k+1}y_{k})\right|,$$ (1)

here, *A*_*i*_ represents the area of a Voronoi cell *i*, *K* is the number of vertices of the polygon, and $\left(x_{k},y_{k}\right)$ denotes the coordinates of the *k*-th vertex, while $\left(x_{k+1},y_{k+1}\right)$ represents the coordinates of the next vertex in a counterclockwise order. The summation iterates over all vertices, and for the last vertex $\left(x_{K},y_{K}\right)$, the first vertex $\left(x_{1},y_{1}\right)$ is used to ensure the polygon is properly closed.

#### Assessing distribution inequality using the concentration index.

The Concentration Index (CI) is a widely used measure to assess the extent of distributional inequality across different socioeconomic groups or regions [23–25]. It quantifies how evenly a specific resource, such as general hospitals and clinics in this study, is distributed relative to an equality benchmark. The CI is derived from the concentration curve, which plots the cumulative percentage of the population (sorted in ascending order by the number of resources, *x*-axis) against the cumulative percentage of the resource (*y*-axis) (see S2 Fig).

As shown in S3 Fig, the dashed black diagonal line indicates perfect equality (i.e., equality line), while the gray curve represents the actual distribution of resources (i.e., concentration curve). The red area, *A*, in S3 Fig indicates the extent to which resources are concentrated in population groups with a higher number of facilities, while the blue area, *B*, represents the degree of resource scarcity in population groups with fewer facilities. The conventional concentration index, ${\text{CI}}_{\text{conv.}}$, is defined as the difference between the two areas, *A* and *B*, located between the concentration curve and the equality line, normalized by the total area under the equality line. Mathematically, it is expressed as $CI_{\text{conv.}}=\frac{\text{Area }B-\text{Area }A}{0.5}$.

The conventional concentration index, ${\text{CI}}_{\text{conv.}}$, ranges from –1 to 1, where 0 indicates perfect equality. Positive values indicate a lack of resources among resource-scarce groups, while negative values signify a concentration of resources among resource-abundant groups. Unlike the Gini coefficient, ${\text{CI}}_{\text{conv.}}$ provides both the magnitude and direction of the inequality. Specifically, it indicates whether resources are disproportionately concentrated among advantaged or disadvantaged groups, such as those defined by income or resource allocation.

However, in this study, to improve interpretability, the sign of ${\text{CI}}_{\text{conv.}}$ is reversed: a positive value indicates the distribution of healthcare resources favoring disadvantaged groups, while a negative value reflects their concentration among advantaged groups. This is achieved by redefining the equation, as shown in Eq 2.

$$CI=\frac{\text{Area }A-\text{Area }B}{0.5}=-{\text{CI}}_{\text{conv.}}.$$ (2)

This approach enables a more nuanced interpretation of the metric, with negative values indicating a greater scarcity of resources across a broad range of population groups. Additionally, subscripts are applied to the CI measure to differentiate hospital types (e.g., public or private, general hospitals or clinics) and to indicate whether population is considered (e.g., population-based; pop, or non-population-based; non). For example, the CI measure for public hospitals without population consideration is denoted as ${\text{CI}}_{\text{public},\text{non}}$.

Note that ${\text{CI}}_{\text{public},\text{non}}=0$ in Sejong, Jeju, and Ulsan in the analysis. In Sejong, ${\text{CI}}_{\text{public},\text{non}}$ is undefined because the city consists of a single administrative district, making distribution measurement impossible. In Ulsan, the absence of public general hospitals results in ${\text{CI}}_{\text{public},\text{non}}=0$. Meanwhile, in Jeju, public hospitals are evenly distributed across its administrative districts, leading to a balanced allocation.

By using the defined CI index, we examine difference between ${\text{CI}}_{\text{non}}$ and ${\text{CI}}_{\text{pop}}$ as ΔCI as shown in Eq 3:

$$\Delta \text{CI}=\frac{{CI}_{\text{non}}-{CI}_{\text{pop}}}{{CI}_{\text{non}}}.$$ (3)

As the ${\text{CI}}_{\text{pop}}$ value becomes more equal, approaching zero, ΔCI approaches 1 regardless of ${\text{CI}}_{\text{non}}$, indicating a 100% improvement in resource equality when accounting for population. The sign of CI indicates whether the resource distribution has improved (positive) or become more unequal (negative). For instance, when ΔCI=−1, the negative sign indicates a worsening of equality when population is considered, and |ΔCI|=1 signifies that the extent of this deterioration is 100% relative to ${\text{CI}}_{\text{non}}$.

#### Quantifying distributional heterogeneity using the entropy index.

The entropy index, *H*, derived from information theory, quantifies the degree of heterogeneity in the distribution of healthcare institutions across administrative regions. It is calculated as $H=-\sum_{i=1}^{N}p(x_{i})\log_{b}p(x_{i})$, where *p*(*x*_*i*_) denotes the proportion of the institutions in a municipal region *i*, *N* is the total number of regions within metropolitan or provincial-level areas, and *b* is the logarithm base. In this study, we applied *b* = 2. To enable comparison across different datasets, the entropy index is normalized as Eq 4:

$$H_{\text{normalized}}=\frac{H}{\log_{b}N},$$ (4)

resulting in values ranging from 0 (complete concentration in one region) to 1 (uniform distribution across all regions).

#### Scaling exponents of public and private healthcare facilities.

Previous research has highlighted key differences in the distribution of commercial and public facilities using an economic model that balances the benefits of facilities with the social opportunity costs of the population [16]. The study found that the relationship between population density *ρ* and facility density *D* follow a simple scaling law, $D∼\rho^{\alpha }$, where the exponent *α* depends on the type of facilities, whether public or commercial. In particular, by comparing empirical data from various facilities in the U.S. and South Korea, it was found that public and commercial facilities follow distinct power-law exponents: $\alpha ∼\frac{2}{3}$ for public facilities and similar to or greater than 1 for commercial facilities. Since different exponents reflect distinct optimization processes of the facilities based on their purpose—$\alpha ∼\frac{2}{3}$ indicates that facilities are placed to optimize the cost of population access, while α≥1 demonstrates a preference for economic benefits, concentrating facilities in highly populated areas—we assess the exponents for public and private general hospitals and clinics across medical specialties to determine whether different types of medical institutions follow distinct optimization strategies.

## Results

We first analyze the regional distribution patterns of public and private general hospitals to examine whether distribution patterns differ by establishment type across various administrative levels. As shown in Fig 1(a) and 1(b), the distribution patterns of private and public general hospitals exhibit distinct characteristics. Private ones are concentrated in five metropolitan areas—(1) Seoul, (2) Daejeon, (3) Daegu, (7) Gwangju, and (5) Busan—whereas public hospitals are more evenly distributed, extending into smaller cities and mid-sized towns. As a result, public hospitals cover relatively larger areas per Voronoi cell, whereas private hospitals are concentrated in smaller regions, with an average Voronoi cell area of 684.85 ${\text{km}}^{2}$ per facility, compared to 1,648.75 ${\text{km}}^{2}$ for public hospitals (Fig 1(a) and 1(b)). This disparity in Voronoi cell areas is further quantified by the Gini coefficient, which indicates a more uneven distribution of private hospitals (Gini coefficient = 0.64), significantly higher than that of public hospitals (Gini coefficient = 0.45) (Fig 1(c) and 1(d)). This gap implies that private hospitals are disproportionately clustered, leaving large areas with minimal access, while public hospitals provide broader regional coverage.

**Fig 2:**
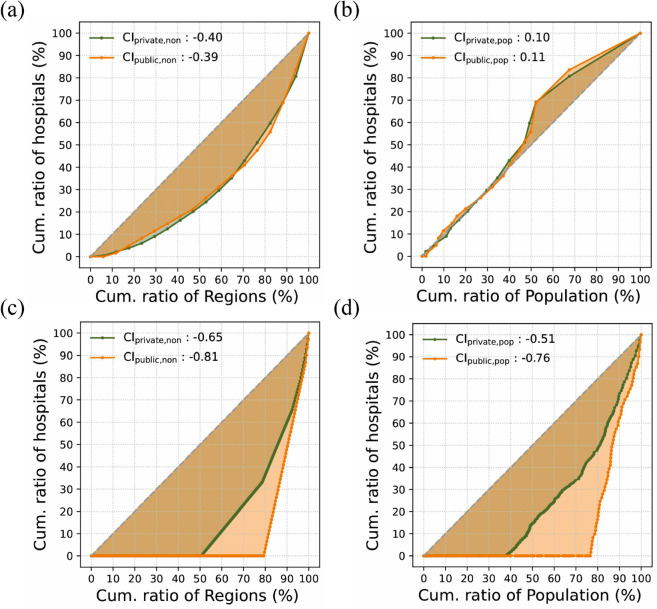
Concentration curve of general hospitals at the provincial and municipal levels (Private vs. Public). The *x*-axis represents the cumulative percentage of provincial regions in (a) and (c), and the cumulative percentage of the population in (b) and (d), with both regions and populations sorted in ascending order by the number of facilities. The *y*-axis shows the cumulative percentage of total facilities. Colors indicate the establishment types of general hospitals: ${\text{CI}}_{\text{private}}$ (green) and ${\text{CI}}_{\text{public}}$ (orange). Panels (a) and (b) show CI at the provincial level, while panels (c) and (d) present CI at the municipal level.

However, these spatial inequality patterns may change when accounting for population distribution. To examine this, we compared the concentration index adjusted for population with the index that does not account for population (see Methods). At provincial scale, Fig 2(a) shows that the ${\text{CI}}_{\text{non}}$ values for both private and public general hospitals are similar, suggesting that their distribution remains balanced at a broad regional level when population is not taken into account.

In contrast to this, at the municipal level without accounting for population Fig 2(c) reveals a significant disparity in the CIs between private and public general hospitals. Both hospital types at the municipal scale have lower CI values than at the provincial level, but the decline is more pronounced for public general hospitals, with ${\text{CI}}_{\text{public},\text{non}}$ decreasing from –0.39 to –0.81 (Fig 2(a) and 2(c)). This is because public general hospitals are more sparsely distributed across cities, leaving more municipalities without them, resulting in a more uneven distribution compared to private general hospitals at the municipal level.

When population is taken into account, the CI values for private and public general hospitals at the provincial level improve closer to zero (${\text{CI}}_{\text{private},\text{pop}}=0.10$, ${\text{CI}}_{\text{public},\text{pop}}=0.11$), showing similar values to each other, as shown in Fig 2(b). This improvement suggests that population density correlates the distribution of general hospitals at the provincial level, regardless of their establishment type. However, Fig 2(b) also shows that the resource-scarce 50% of the population has marginal access to general hospitals within their provincial regions, aligning closely with the equality line, while the resource-abundant 50% experiences an oversupply, with facilities disproportionately concentrated relative to the population.

At the municipal level, ${\text{CI}}_{\text{pop}}$ shows improvement over ${\text{CI}}_{\text{non}}$ for both types of general hospitals, indicating that population density plays a crucial role in facility distribution even at finer administrative scales. However, the disproportionate concentration of general hospitals remains evident (Fig 2(d)). Contrary to expectations, at the municipal level around 80% of resource-scarce regions lack any public general hospital in their jurisdiction, indicating an uneven distribution even compared to private hospitals (Fig 2(d)).

**Fig 3:**
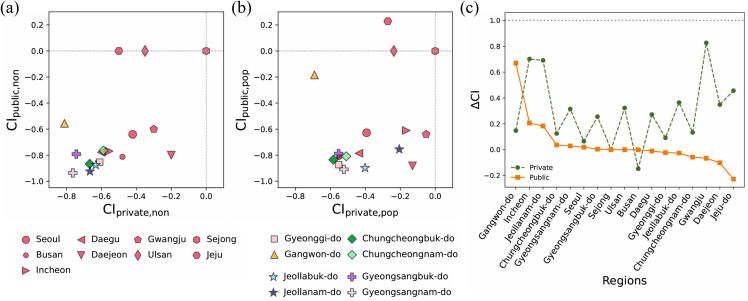
Private and public CI values for general hospitals at the provincial level. The panels (a) and (b) illustrate ${\text{CI}}_{\text{private}}$ and ${\text{CI}}_{\text{public}}$ for general hospitals in provincial-level regions, both without and with population consideration, respectively. Different colors and markers represent distinct provincial-level regions in South Korea. Panel (c) shows ΔCI for private (green circles) and public (orange squares) general hospitals in provincial-level regions with population consideration. Dashed and solid lines are provided as visual guidelines for readers.

To understand a city’s distribution patterns of general hospitals, both public and private CI values should be considered simultaneously. To achieve this, we examined the CI values of public and private hospitals for each provincial region. In general, ${\text{CI}}_{\text{private}}$ distinguishes more clearly between metropolitan and non-metropolitan areas than ${\text{CI}}_{\text{public}}$, highlighting the concentration gap of private general hospitals between metropolitan regions and other cities (Fig 3(a)). Specifically, in Fig 3(a), most metropolitan cities (red) have ${\text{CI}}_{\text{private},\text{non}}$ in the range of [–0.6,0.0], whereas non-metropolitan regions show ${\text{CI}}_{\text{private},\text{non}}\in [-0.6,-0.8]$. Although not as pronounced as in the private sector, the ${\text{CI}}_{\text{public}}$ values are still slightly lower in non-metropolitan cities than in metropolitan cities. This indicates that when considering only administrative divisions, the distribution of general hospitals appears to vary depending on whether an area is designated as a metropolitan city in both the public and private sectors (Fig 3(a)).

However, when accounting for population, most regions exhibit an improvement in ${\text{CI}}_{\text{pop}}$, particularly with a reduced disparity in ${\text{CI}}_{\text{private},\text{pop}}$ between metropolitan cities and other regions. Specifically, some regions, such as Incheon, Jeollanam-do, and Gwangju, exhibit a relatively high improvement with ΔCI>0.5 (Fig 3(c)). In contrast, Busan is the only region where the disproportionate distribution is amplified, as indicated by ΔCI<0 (Fig 3(b) and 3(c)). Given that ΔCI<0 signifies a decline in distributional equity for general hospitals when considering population density (Methods), this suggests that a larger portion of Busan’s population is concentrated in areas with limited general hospital resources. These results suggest that population density can be correlated to the distribution of private general hospitals more than administrative regions, with spatial concentration patterns of population outweighing overall population size (Fig 3(b)).

For the public sector, most regions persist low negative CI regardless of population consideration (Fig 3(a) and 3(b)). However, a closer look reveals that the ${\text{CI}}_{\text{public},\text{pop}}$ become more unequal in most regions (ΔCI<0 in Fig 3(c)), suggesting that the distribution of public general hospitals is less sensitive to population concentration.

**Fig 4:**
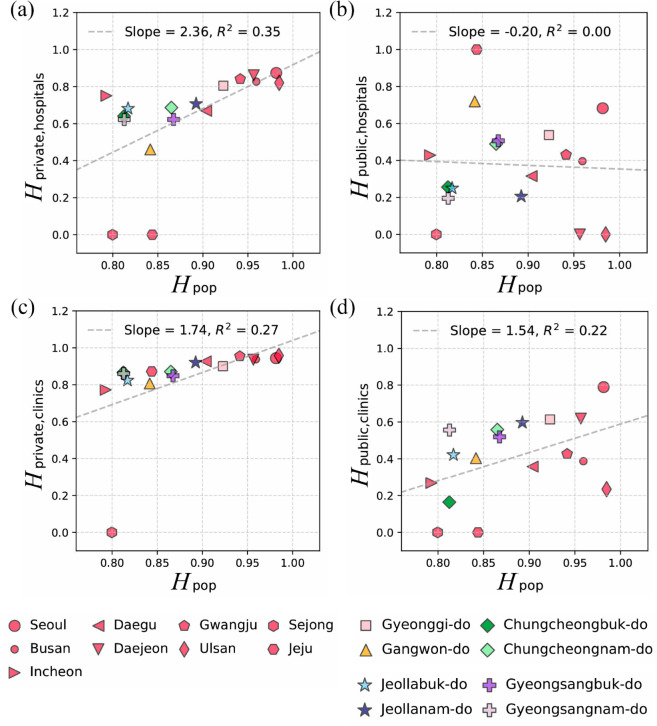
The entropy index *H* of population and medical institution distribution across provincial-level regions. Panels (a) and (b) present the entropy index for general hospitals $H_{\text{hospitals}}$ in relation to $H_{\text{pop}}$ across regions for private and public institutions, respectively, with different colors and markers indicating distinct regions. Panels (c) and (d) display the entropy index for clinics $H_{\text{clinics}}$, which are smaller-scale medical institutions compared to the general hospitals shown in (a) and (b). The dashed line represents the regression line indicating the slopes.

Motivated by the indirect observation that private hospitals can be associated with the heterogeneity of the population distribution rather than the size of the population itself, we examine the correlation between the distribution patterns of the facilities and the heterogeneity of the population with the entropy index *H* (see Methods). Fig 4(a) demonstrates a strong correlation between the entropy of private general hospitals $H_{\text{private, hospitals}}$ and the entropy of population distribution $H_{\text{pop}}$ (*R*^2^ = 0.35), while public general hospitals’ distributional heterogeneity $H_{\text{public, hospitals}}$ exhibits an independent pattern of population entropy (Fig 4(b); *R*^2^ = 0.0).

This correlation between population distribution entropy and the concentration entropy of private hospitals persists in clinics, which are categorized by medical specialties and operate on a smaller scale than general hospitals (Fig 4(c); *R*^2^ = 0.27). Consistent with the pattern observed for private general hospitals, private clinics exhibit a strong correlation with population entropy, whereas public clinics show a weaker association and greater deviations from the regression line (see Fig 4(c) and 4(d)). These results confirm that private healthcare institutions are more responsive to the heterogeneity of population concentration than public facilities, reflecting their distinct distributional principles based on the two types of establishment purposes.

Why does this difference in distributional patterns between the public and private sectors, related to population density, arise? To understand the underlying principles behind these distributional differences, we build on a previous study that identified a power-law relationship between population density and facility density for public and commercial facilities [16]. This study found that public and commercial facilities exhibit distinct power-law exponents (*α*), with public facilities following $\alpha \approx \frac{2}{3}$ and commercial facilities α≈1 (see Methods). Using these established exponents, we examine whether public and private healthcare institutions follow different distributional principles.

**Fig 5:**
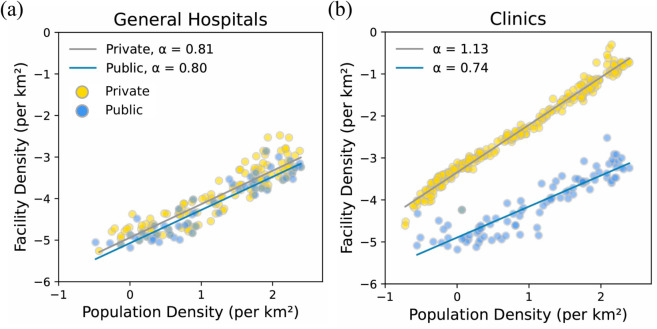
The relationship between facility density and population density for general hospitals and clinics. Panels present a scatter plot of municipal-level population density versus (a) general hospital and (b) clinic facility density on a logarithmic scale. Colors distinguish the establishment types of hospitals and clinics: private (yellow) and public (blue). The slopes, *α*, are estimated using linear regression for private (gray line) and public (blue line).

For general hospitals, both private and public facilities exhibit α≈0.8, which falls between the exponents of the public and private sectors (Fig 5(a)). This suggests that while general hospitals exhibit some characteristics of the public sector, they also incorporate both public and commercial attributes. This intermediate scaling exponent may reflect the hybrid role of general hospitals in South Korea, which balance public policy mandates and market-driven operations [26].

In contrast, clinics show clear distinct patterns based on their purpose of establishment (Fig 5(b)). Private clinics, with α≈1, exhibit commercial-like characteristics by concentrating in highly populated areas, reflecting their market-oriented tendency. In contrast, public clinics, with α=0.74, are more evenly distributed to ensure broader access to healthcare services, reflecting an approach focused on minimizing access costs.

**Fig 6:**
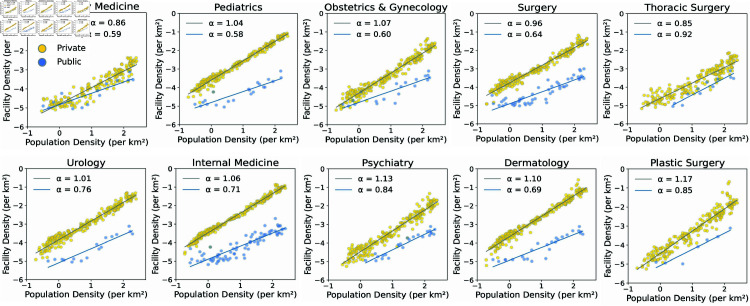
The relationship between public and private clinic density and population density across medical specialties. Each scatter plot illustrates the relationship between population density and clinic density across various medical specialties, with both axes on a logarithmic scale. Private clinics are shown in yellow, while public ones are in blue. Linear regression lines are fitted for both types (public, and private) to estimate the scaling exponent *α*, with private hospitals represented in gray and public hospitals in blue.

Looking more closely, one can observe that the distribution characteristics of clinics vary by medical specialty, as shown in Fig 6. As expected, private clinics generally exhibit *α* values close to 1, indicating evident commercial characteristics, particularly in specialties such as psychiatry (α=1.13), plastic surgery (α=1.17), and dermatology (α=1.10). These high exponents illustrate the commercial orientation of these specialties, as they are predominantly concentrated in densely populated, high-demand areas. In contrast, essential medical specialties such as emergency medicine (α=0.86) and thoracic surgery (α=0.85) show lower *α* values, even in the private sector, suggesting that their distribution cannot be fully explained by commercial motives alone.

Public clinics generally exhibit *α* values closer to $\frac{2}{3}$ or lower, indicating a more even distribution across regions for certain specialties, such as internal medicine (α=0.71) and surgery (α=0.64). This reflects the emphasis of the public health sector on equitable access to healthcare rather than commercial considerations. However, essential specialties such as emergency medicine (α=0.59), pediatrics (α=0.58), and obstetrics and gynecology (α=0.60) exhibit exponents lower than $\frac{2}{3}$, indicating that their distribution falls short of an optimally public-oriented allocation and remains insufficient. This lack of allocation highlights that, although clinics are primary care facilities intended to be easily accessible to most of the population, the distribution of certain specialties fails to fulfill their public purpose.

**Fig 7:**
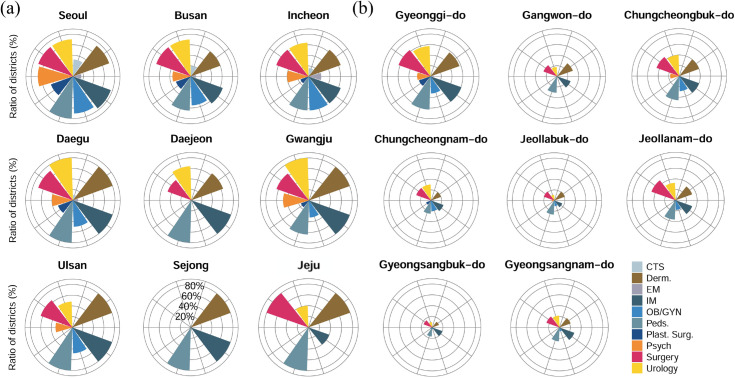
Percentage of regions falling within the equitable ${\text{CI}}_{\text{private},\text{pop}}$ range by medical specialty. The charts show the proportion of municipalities within provincial-level administrative regions where ${\text{CI}}_{\text{private},\text{pop}}\in [-0.4,0.4]$ for private clinics across different medical specialties. Panel (a) illustrates metropolitan regions, while panel (b) represents other provincial-level regions.

As private medical institutions cover smaller areas than public ones (Fig 1), they can play a crucial role in shaping healthcare accessibility in most cities. In particular, having a diverse range of specialties nearby is essential for ensuring healthcare accessibility at the local level (CI values for for municipal-level clinics by medical specialties; see S4 Fig). To quantify this, we assessed distributional equity by specialty using the ratio of municipalities where the ${\text{CI}}_{\text{private},\text{pop}}$ of clinics for each specialty falls within the equitable range of [–0.4,0.4] at the provincial level [27]. The results reveals significant regional and specialty-specific disparities as shown in Fig 7. In most metropolitan regions, more than 60% of municipalities for certain specialties, such as dermatology (Derm.), pediatrics (Peds.), internal medicine (IM), and surgery fall within the equitable range of ${\text{CI}}_{\text{private},\text{pop}}\in [-0.4,0.4]$, suggesting relatively well-developed distribution of a variety of healthcare specialties (Fig 7(a)). In contrast, special administrative regions like Sejong and Jeju exhibit extreme disparities, with only a few specialties having a high percentage of equitable distribution, while many others are scarcely represented.

In non-metropolitan regions, except for Gyeonggi-do—which can practically be considered a metropolitan area near Seoul—most provinces have fewer than 40% of municipalities with an equitable distribution across multiple specialties, indicating severe imbalances in the availability of medical specialties (Fig 7(b)). This disparity is further exacerbated by the fact that some areas have no districts within the equitable range for certain specialties, highlighting the severe shortage and uneven distribution of medical services across regions, as shown in Fig 7(b). In particular, essential specialties such as emergency medicine, obstetrics and gynecology, pediatrics, and thoracic surgery exhibit notably low percentage in non-metropolitan areas, further underscoring the imbalance in the distribution of essential medical services in these regions.

**Fig 8:**
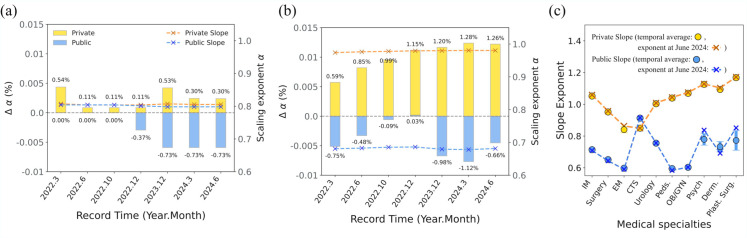
Temporal changes in the scaling exponent *α.* The changes in the scaling exponent (Δα) relative to the December 2021 baseline slope (Bar plot: private (yellow), public (blue); corresponding to the left *y*-axis) and the actual scaling exponent (Dashed lines: private (orange), public (blue); corresponding to the right *y*-axis) for (a) general hospitals and (b) clinics have been tracked from March 2022 to June 2024. Panel (c) presents the temporal changes in clinics by specialty. The temporal average of scaling exponents *α* for each medical specialty is represented by circle markers, while the current scaling exponent *α* (as of June 2024) is highlighted with an ‘x’ marker and dashed lines for private (blue) and public (orange) clinics. Error bars are shown the standard deviations of temporal scaling exponents. Dashed lines are provided as visual guidelines for readers.

So far, the results confirm a clear regional disparity in the current distribution of healthcare institutions, as well as in the distributional principles correlated with their establishment types and medical specialties. However, understanding the evolving tendencies of the scaling exponents of public and private institutions can help identify potential risks in changes to accessibility and equity. To address this, we conducted a time-series analysis of the hospital’s scaling exponent (*α*) from March 2022 to June 2024. As shown in Fig 8(a), private general hospitals exhibited relatively stable *α* values around 0.80 over time. The exponent of public general hospitals exhibited similar values to that of private ones but has shown a continuous decrease since December 2022 compared to December 2021.

For the scaling exponents of clinics in Fig 8(b), *α* values gradually increased for private clinics, indicating a slow but steady shift toward a more commercial-like distribution pattern. This suggests that private clinics may gradually align with commercial tendencies over time, though the pace of change remains moderate. In contrast, public clinics exhibit fluctuations in *α* but maintain a relatively stable scaling pattern, with α≈0.68 persisting through 2024.

However, the stable distribution patterns of the public clinics vary across medical specialties, with little difference between the current scaling exponent (as of June 2024) and the two-year average (Fig 8(c)). Notably, when examining essential specialties such as emergency medicine, pediatrics, and obstetrics and gynecology, public clinics for these specialties exhibit $\alpha <\frac{2}{3}$, indicating a sparser distribution than the theoretically expected value of $\frac{2}{3}$ (Fig 8(c)). In particular, the scaling characteristics of public pediatrics, emergency medicine, and surgery have become sparser over time, as shown in S5 Fig. Even in the private sector, specialties such as emergency medicine and thoracic surgery display relatively low concentrations, indicating a lack of motivation to maintain these essential specialties in both public and private sectors.

## Conclusion and discussion

In this study, we examined the regional distribution patterns of public and private healthcare institutions, taking medical specialties into account. From the results, we found that while public hospitals are more widely distributed than private hospitals, many areas still lack public hospitals, resulting in greater inequality when analyzed at a more granular regional level.

These disparities in distribution become even more pronounced when establishment purpose is considered alongside population factors. For instance, private general hospitals exhibit a clearer distinction between metropolitan and other regions in terms of concentration index (CI) when population is not taken into account. However, the correlation between population and the facility entropy index suggests that these differences may be more closely associated with variations in population distribution patterns than with absolute population size. This is particularly evident in Busan, where a large population size does not necessarily lead to a more equitable distribution of healthcare facilities, further reinforcing the importance of spatial population concentration patterns in shaping institutional distribution.

In terms of the diverse and equitable distribution of medical specialties, the proportion of municipalities with an equitable CI level reveals clear regional differences, particularly between metropolitan and non-metropolitan areas. The results demonstrate that most metropolitan regions provide citizens with diverse medical accessibility across various medical services, whereas other regions face a lack of diversity in healthcare availability. If the distribution of public hospitals remains limited and the commercial-like tendencies of private hospitals intensify over time, as observed in the temporal evolution of distribution exponents, the gap in medical accessibility between metropolitan and non-metropolitan regions may further expand. These findings may serve as a reference for ongoing discussions in South Korea on improving healthcare accessibility and reducing regional disparities [28–30].

The analysis of the scaling exponent between population density and facility density provided insights into the underlying distributional principles of public and private healthcare institutions across medical specialties. For general hospitals, which exhibit a hybrid characteristic of both public and private facilities, this pattern can be better understood in light of the institutional context in South Korea. In the South Korean healthcare system, general hospitals often fulfill dual objectives—supporting public health strategies, while maintaining fee-for-service operations under the National Health Insurance system. This mixed public–private operational model is explicitly recognized in WHO’s Health System Review of Korea as combining equity goals with financial imperatives [26]. Such institutional hybridity likely underlies the intermediate scaling response we observed.

As expected, private healthcare facilities—similar to commercial establishments such as coffee shops [16]—show a stronger association with population distribution patterns, which may relate to economic considerations more than in public institutions. This pattern is particularly evident in certain medical specialties, such as plastic surgery, dermatology, and psychiatry, while it is relatively subdued in essential departments like thoracic surgery, emergency medicine, pediatrics, and obstetrics and gynecology. Furthermore, the sparse distribution of essential departments in both public and private medical sectors raises concerns about disparities in primary healthcare accessibility, as well as the structural factors—such as policy interventions and resource allocation—required to improve essential healthcare services in underserved regions.

Building on this, our findings suggest that targeted policy initiatives—such as reinforcing essential specialties (e.g., pediatrics, obstetrics, emergency medicine) in public hospitals located in non-metropolitan regions—may help mitigate regional disparities. This aligns with recent government policy efforts aimed at strengthening essential healthcare services in non-metropolitan areas through targeted public-sector investment [31,32].

Despite its contributions, this study has certain limitations. This analysis is based on administrative districts, which may not fully reflect the actual distribution of accessible healthcare institutions within and across cities. For example, a hospital located in a neighboring district may be more accessible to certain populations than those within their designated administrative boundary. To address this, future studies could consider using living areas rather than administrative boundaries for a more precise assessment. In addition, future research should take a more refined approach by considering factors such as hospital bed capacity, treatment volume, and the number of physicians, along with their distribution across medical specialties.

Incorporating these variables would enable a more precise identification of regions with insufficient medical resources and provide deeper insights into disparities in medical institutions and healthcare accessibility. Furthermore, integrating geographic factors and refining measurement indicators will strengthen efforts to address regional healthcare disparities, ultimately contributing to more equitable access to medical services for all. Building on this, policy interventions informed by our findings may help reduce avoidable mortality and promote more equitable health outcomes nationwide.

Although this study focuses on South Korea, the methodological framework—particularly the integration of spatial concentration indices and scaling analysis—may be applicable to other countries experiencing urban–rural disparities in healthcare access. In this regard, we expect that the current quantification of regional healthcare disparities, which considers establishment purpose, various types of healthcare facilities, and medical specialties, will provide a foundation for future research in this direction.

## Supporting information

S1 Fig Provincial and municipal administrative regions in South Korea. (PDF)

S2 Fig Effect of population–facility distribution relationships on spatial concentration indices. (PDF)

S3 Fig An example for a concentration curve. (PDF)

S4 Fig Comparison of CI at the municipal level for clinics across medical specialties (private vs. public). (PDF)

S5 Fig Temporal changes in the scaling exponent *α* by medical specialty. (PDF)
