# Supplementary figures and images for: Regional disparities in the distribution of public and private healthcare facilities in South Korea
Source: PLoS One. 2025 Sep 24;20(9):e0330090. doi: 10.1371/journal.pone.0330090 (PMC12459777; doi:10.1371/journal.pone.0330090)

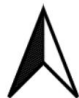

### Legend

- 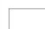 sigungu
- 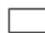 sido

0 50 100 150 km

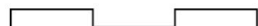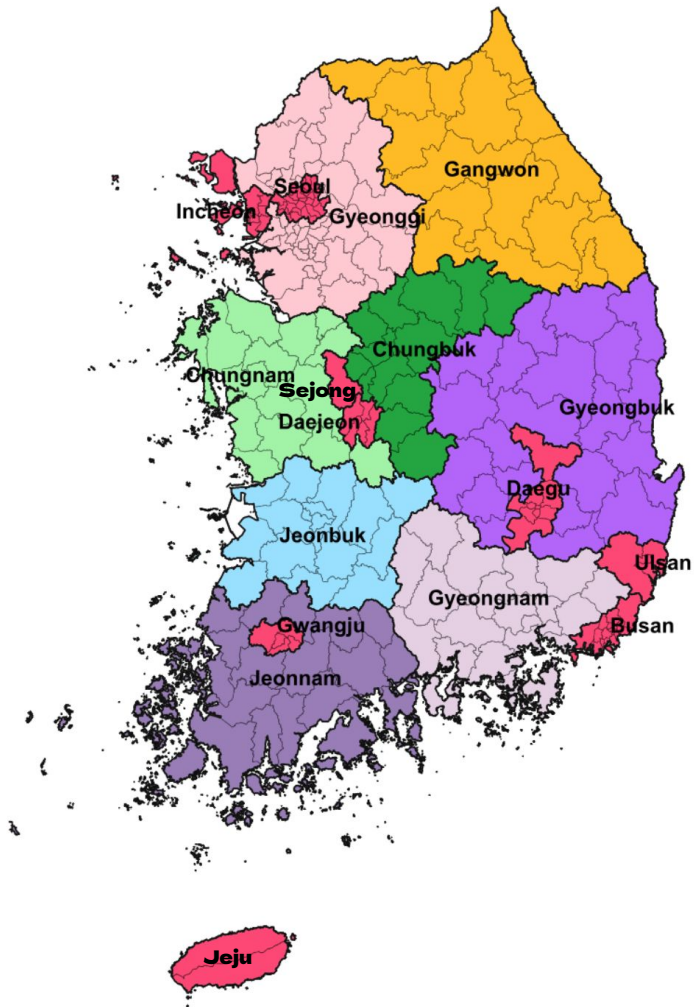

Supplement: S1 Fig — (PDF) [file pone.0330090.s001.pdf]

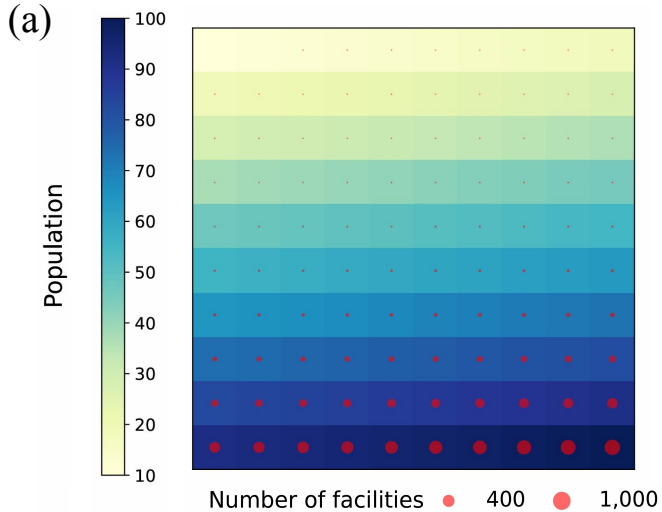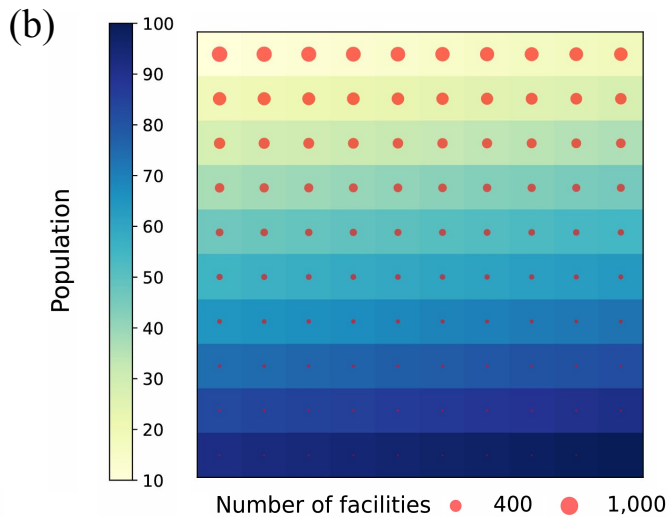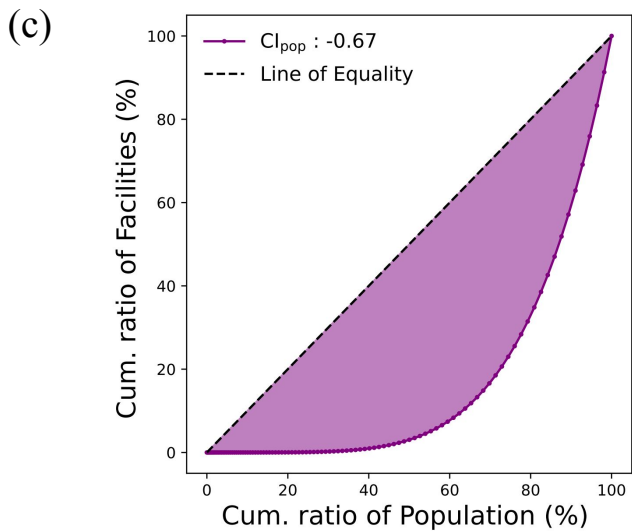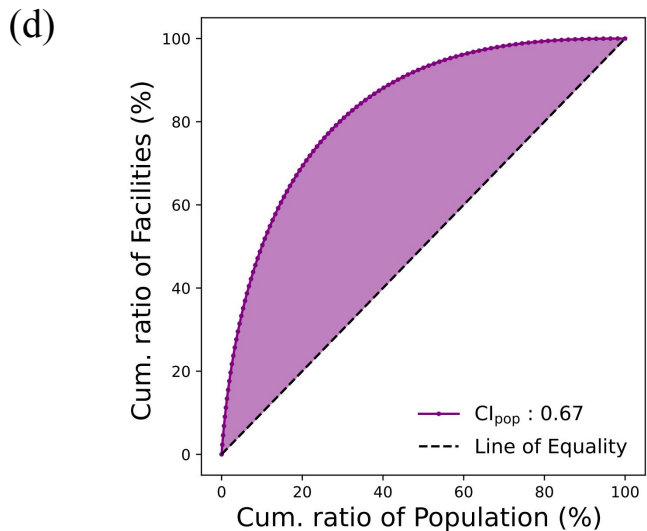

Supplement: S2 Fig — (PDF) [file pone.0330090.s002.pdf]

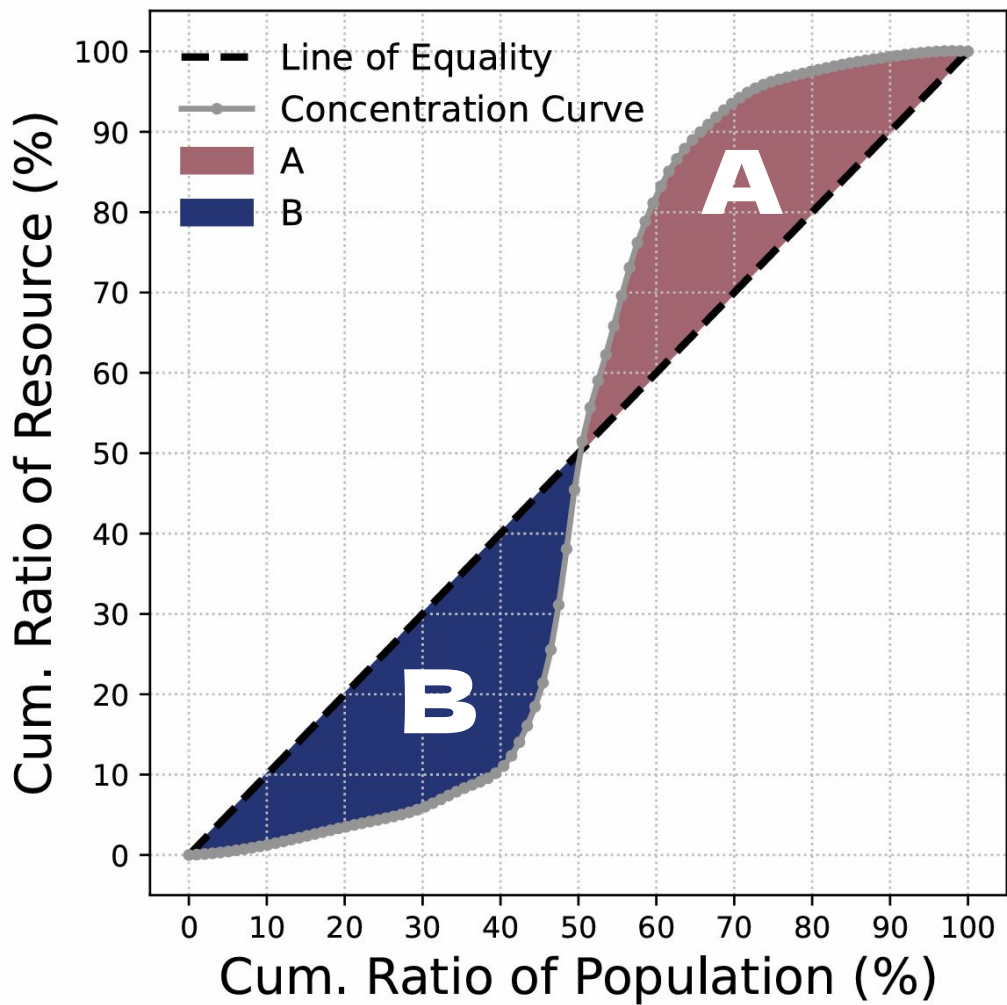

Supplement: S3 Fig — (PDF) [file pone.0330090.s003.pdf]

# municipal-level

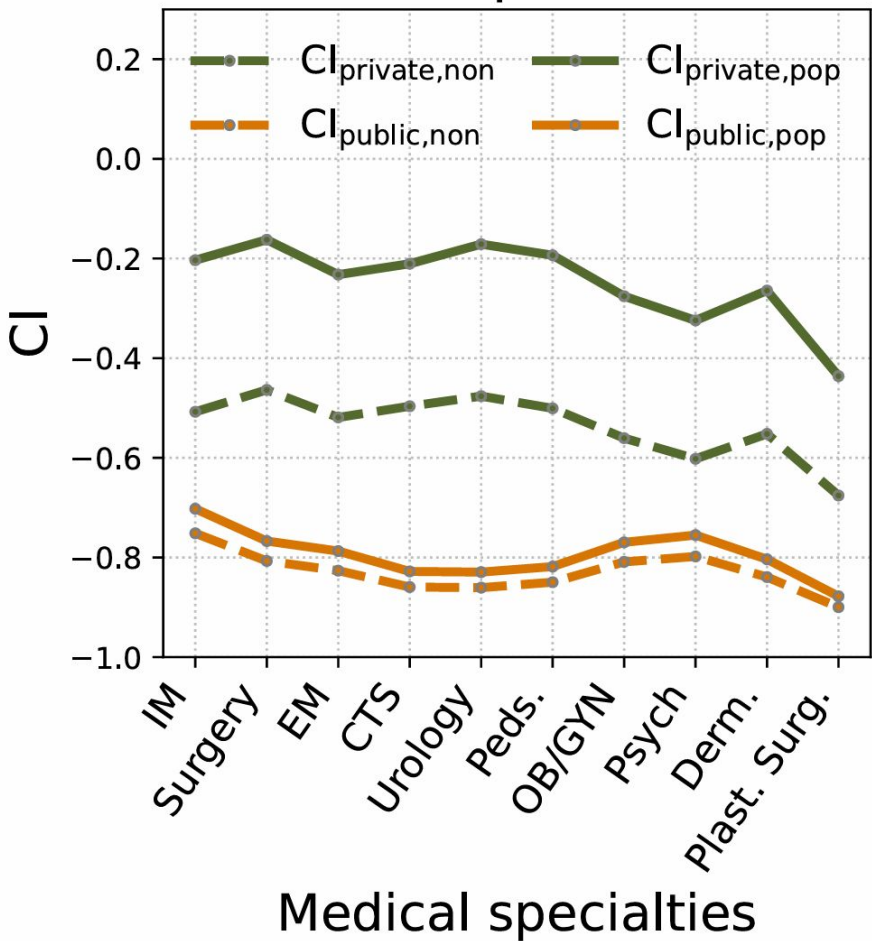

Supplement: S4 Fig — (PDF) [file pone.0330090.s004.pdf]

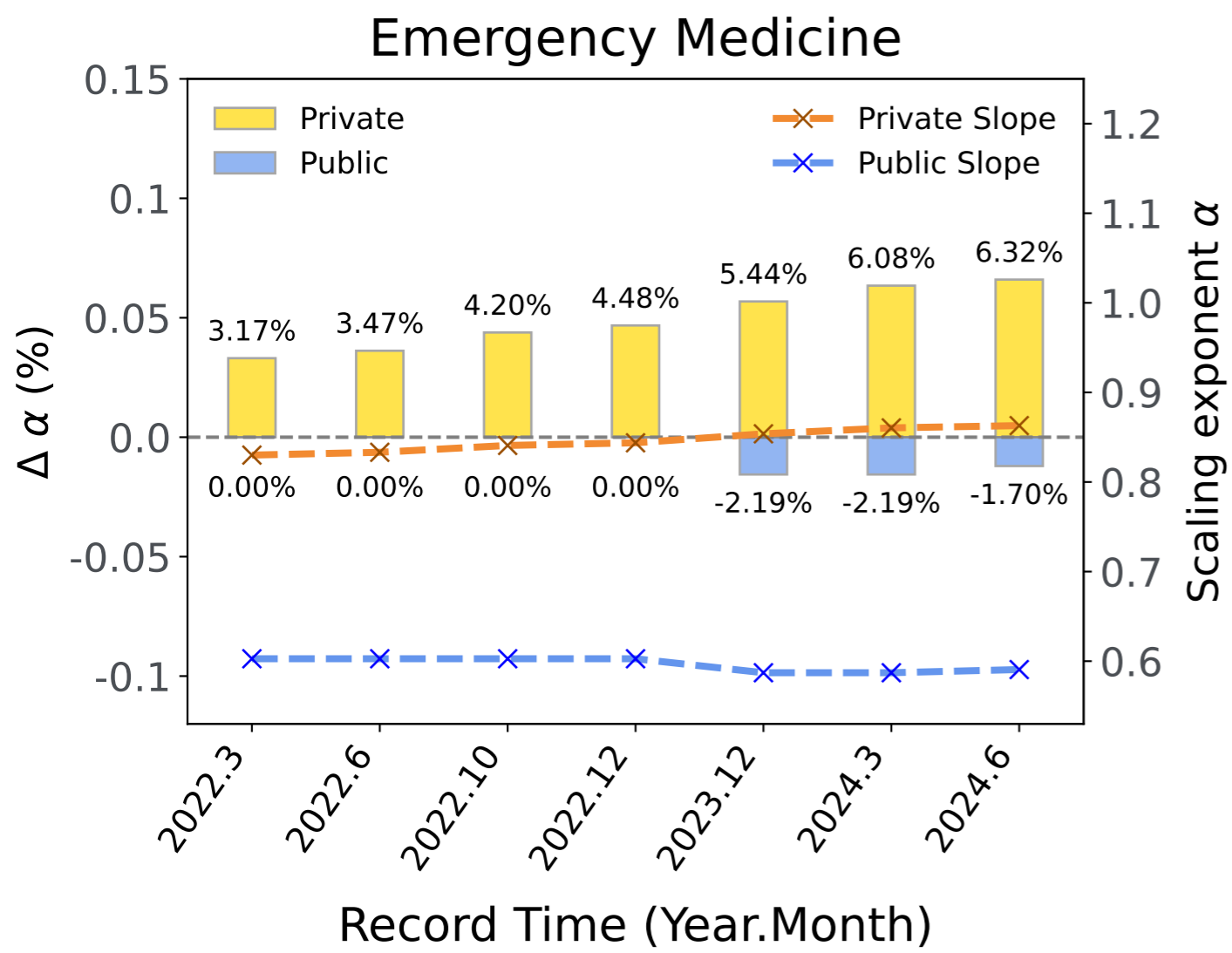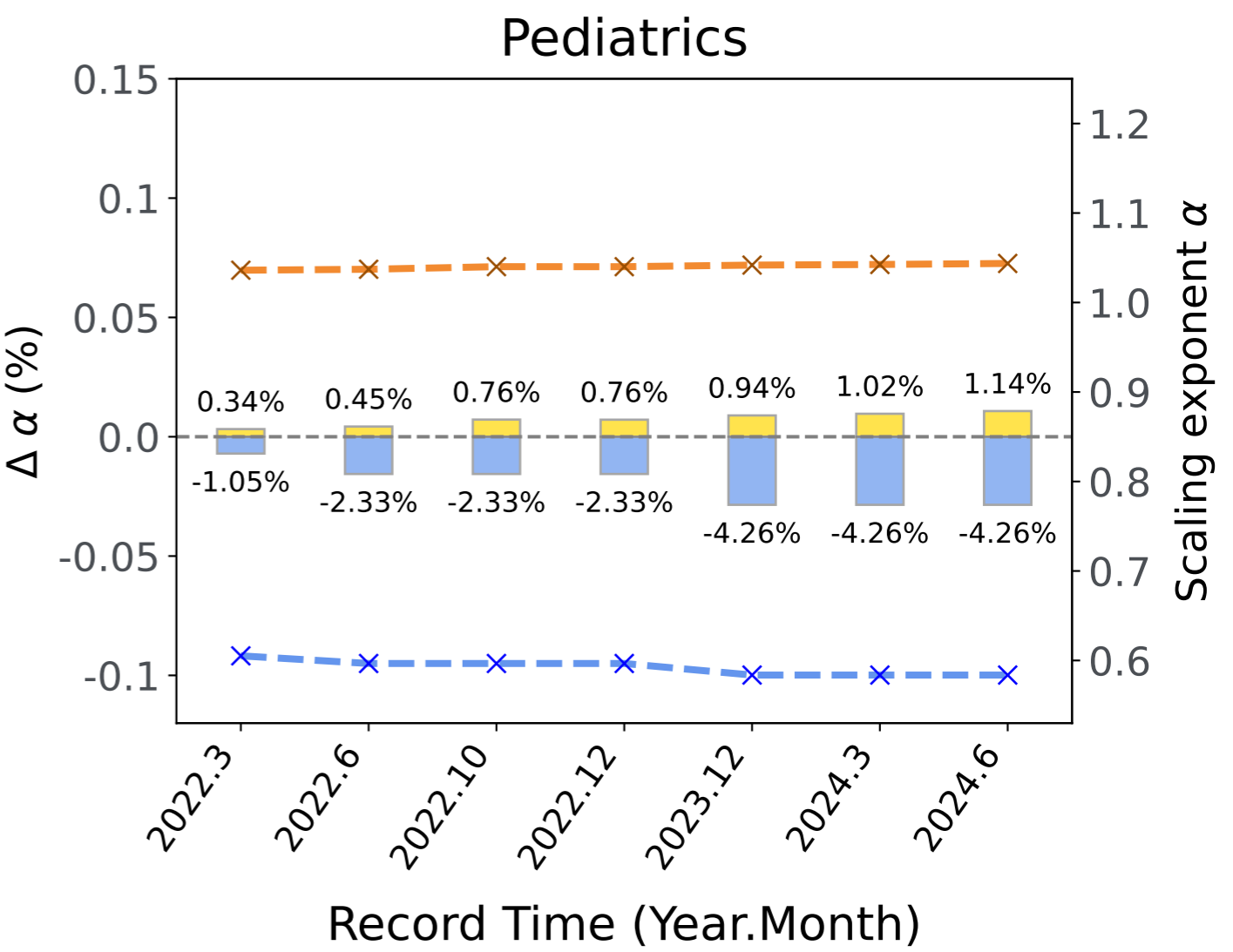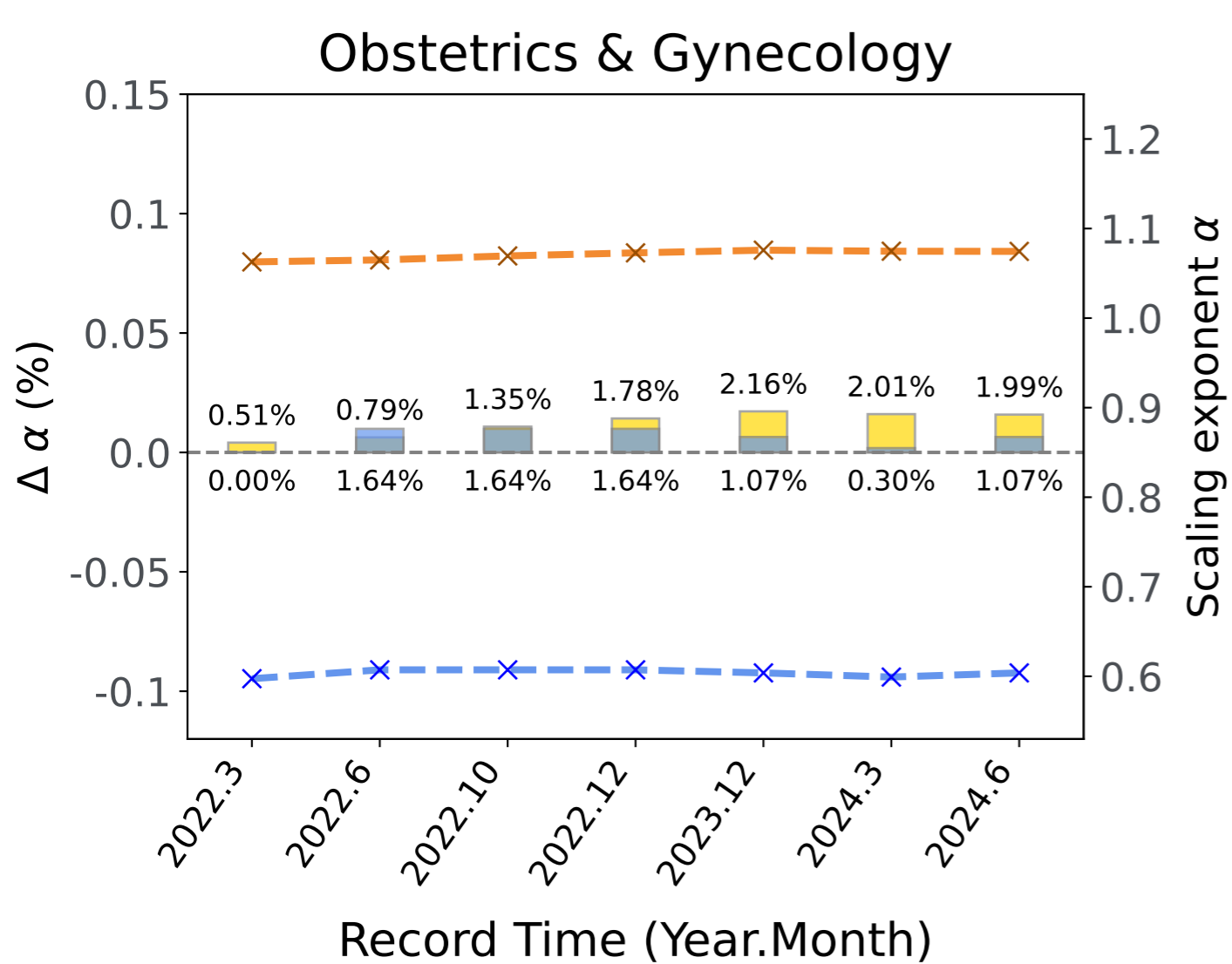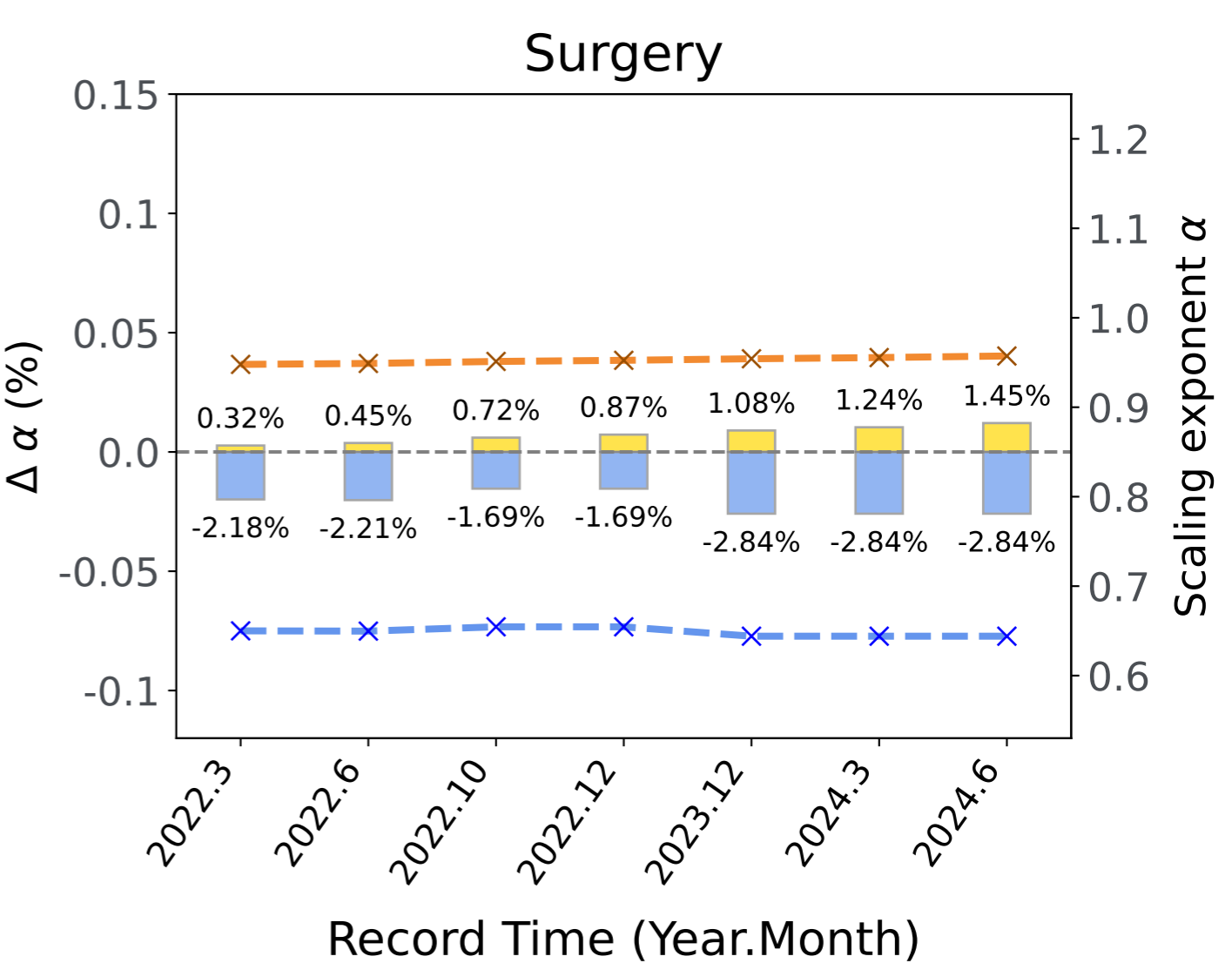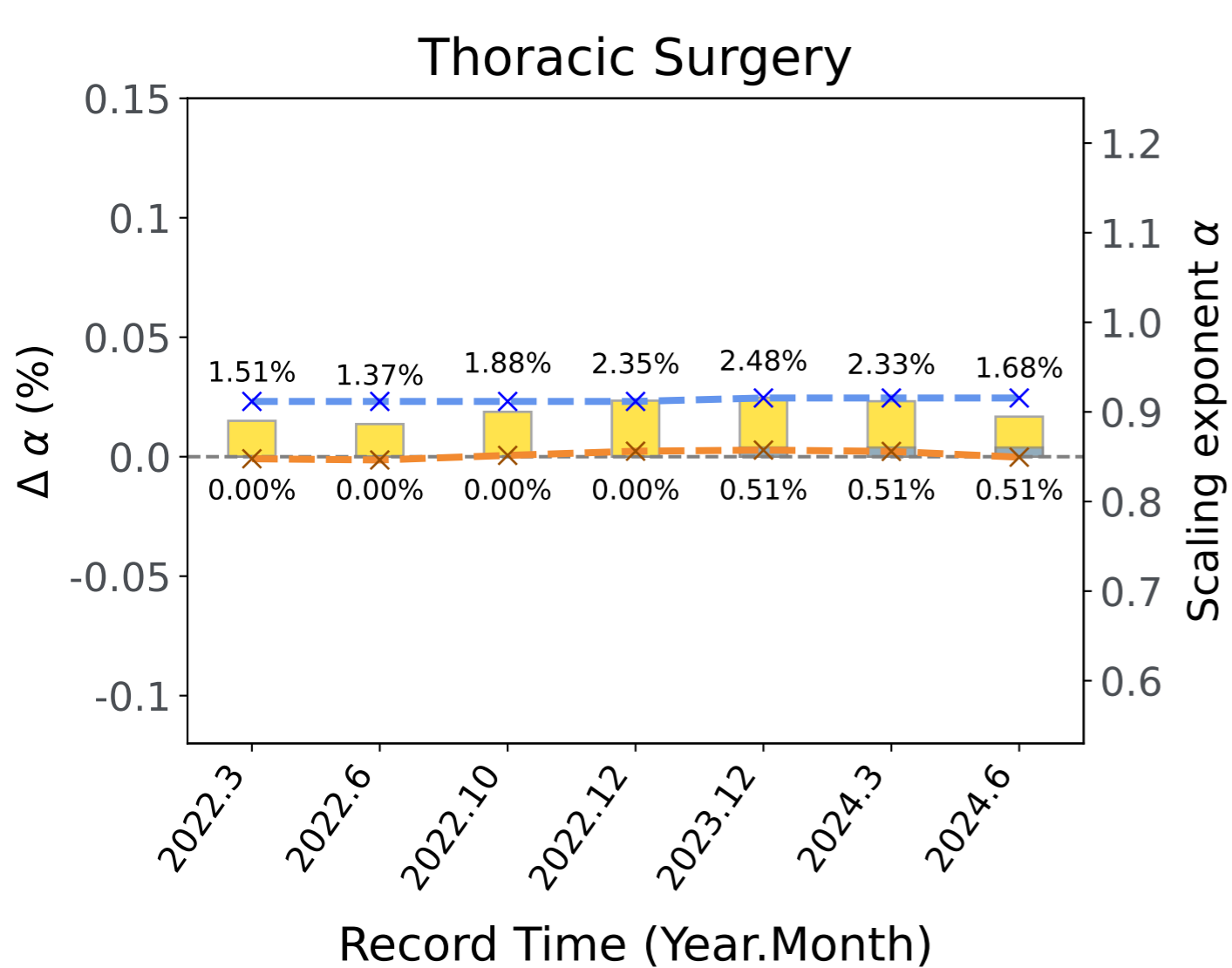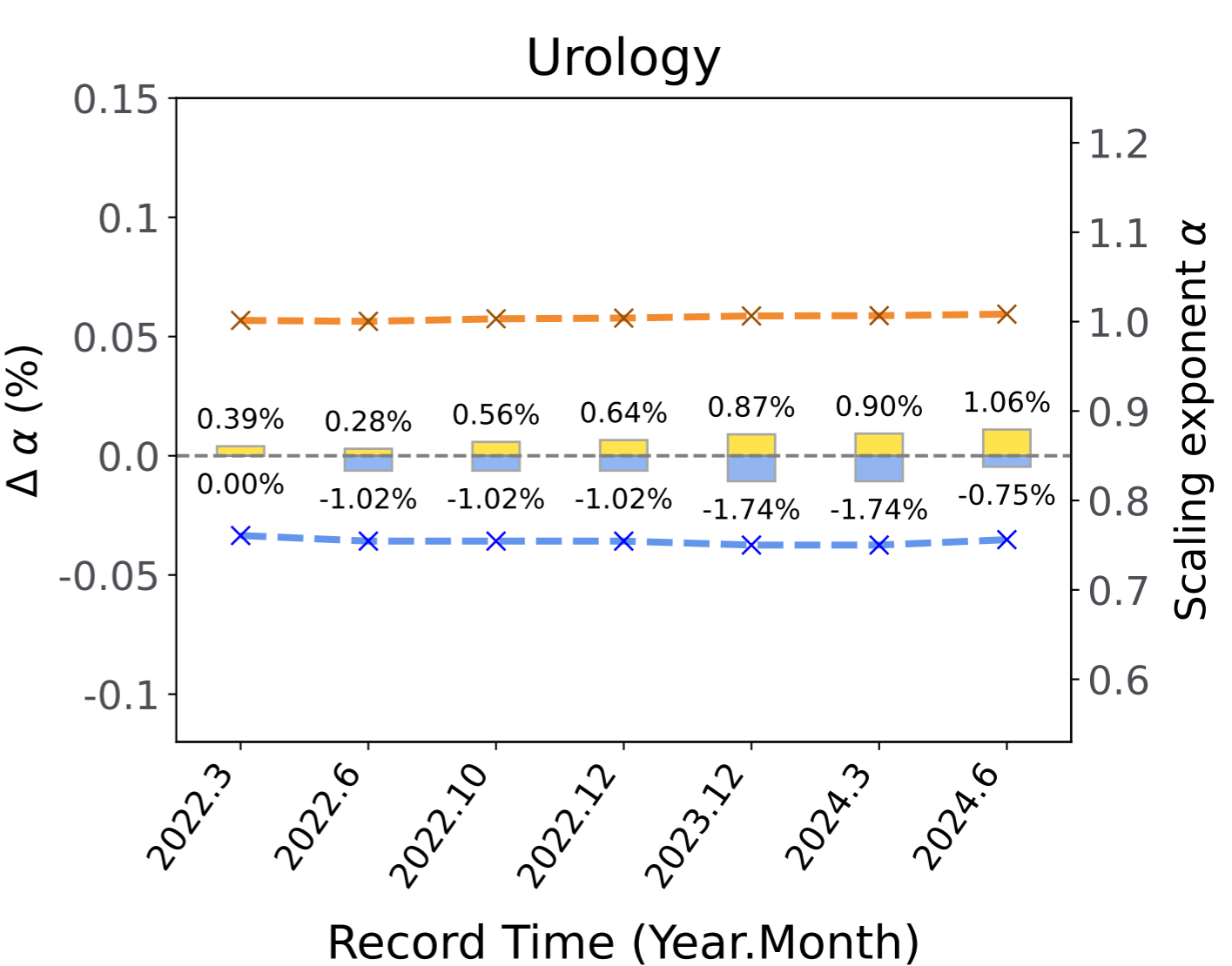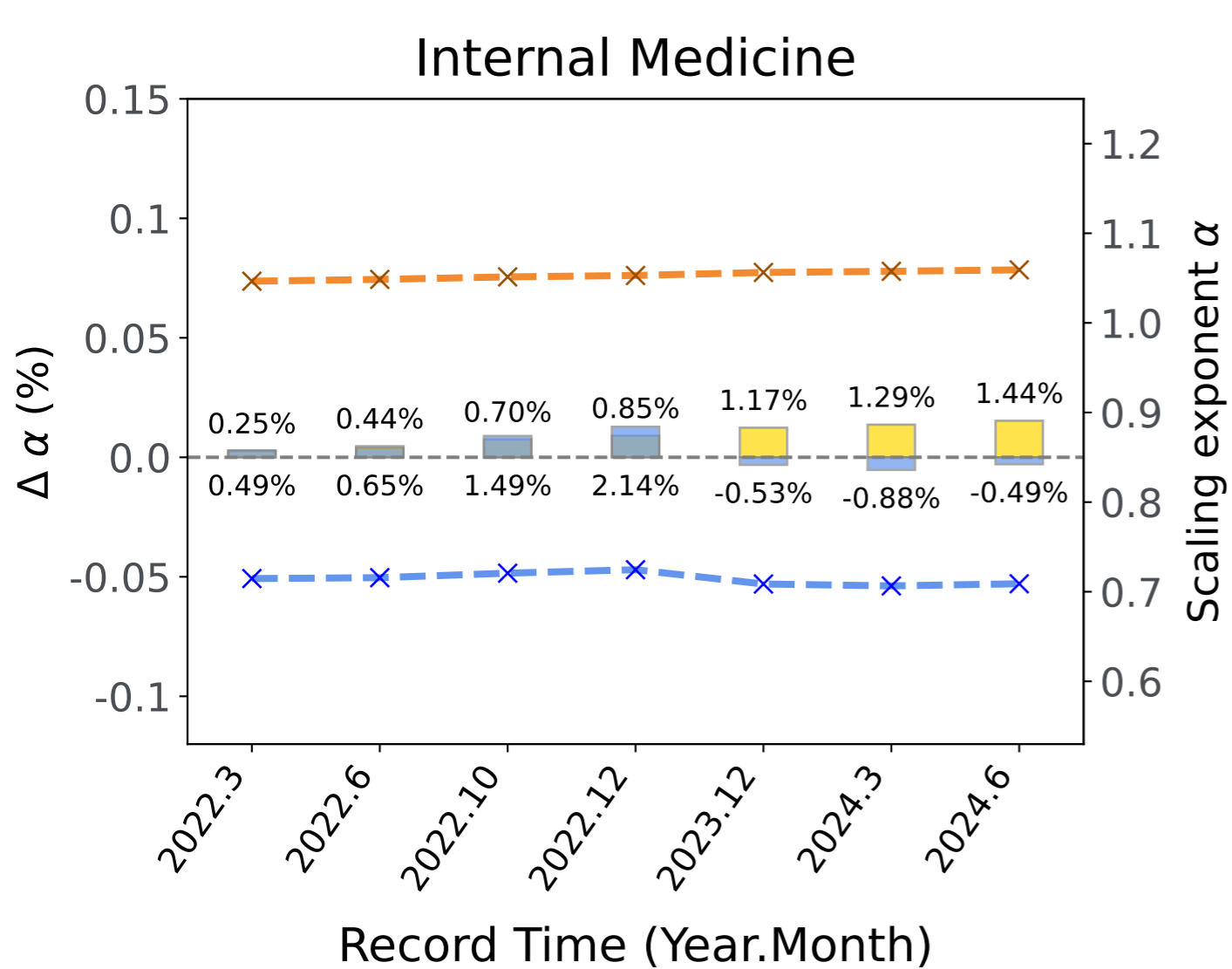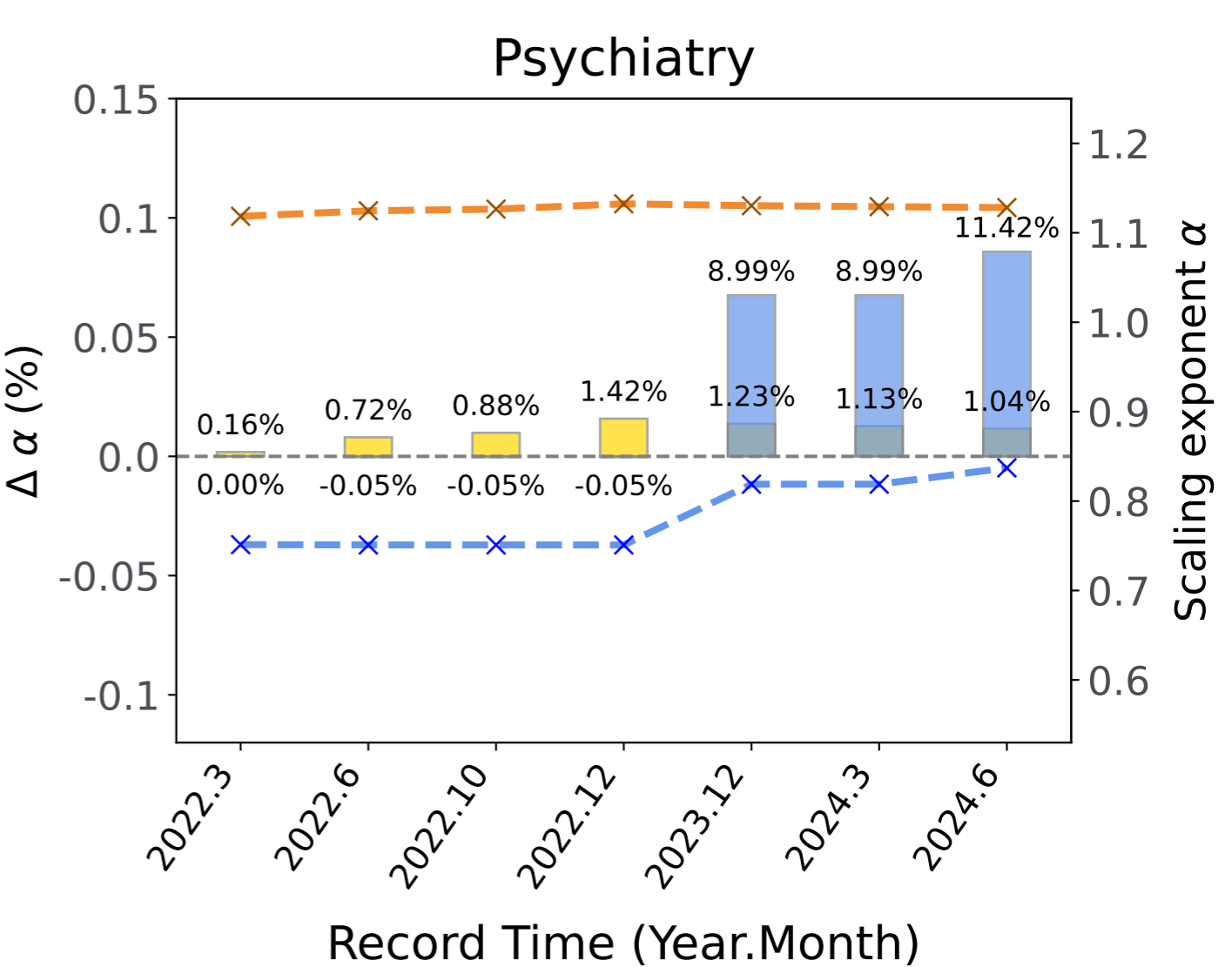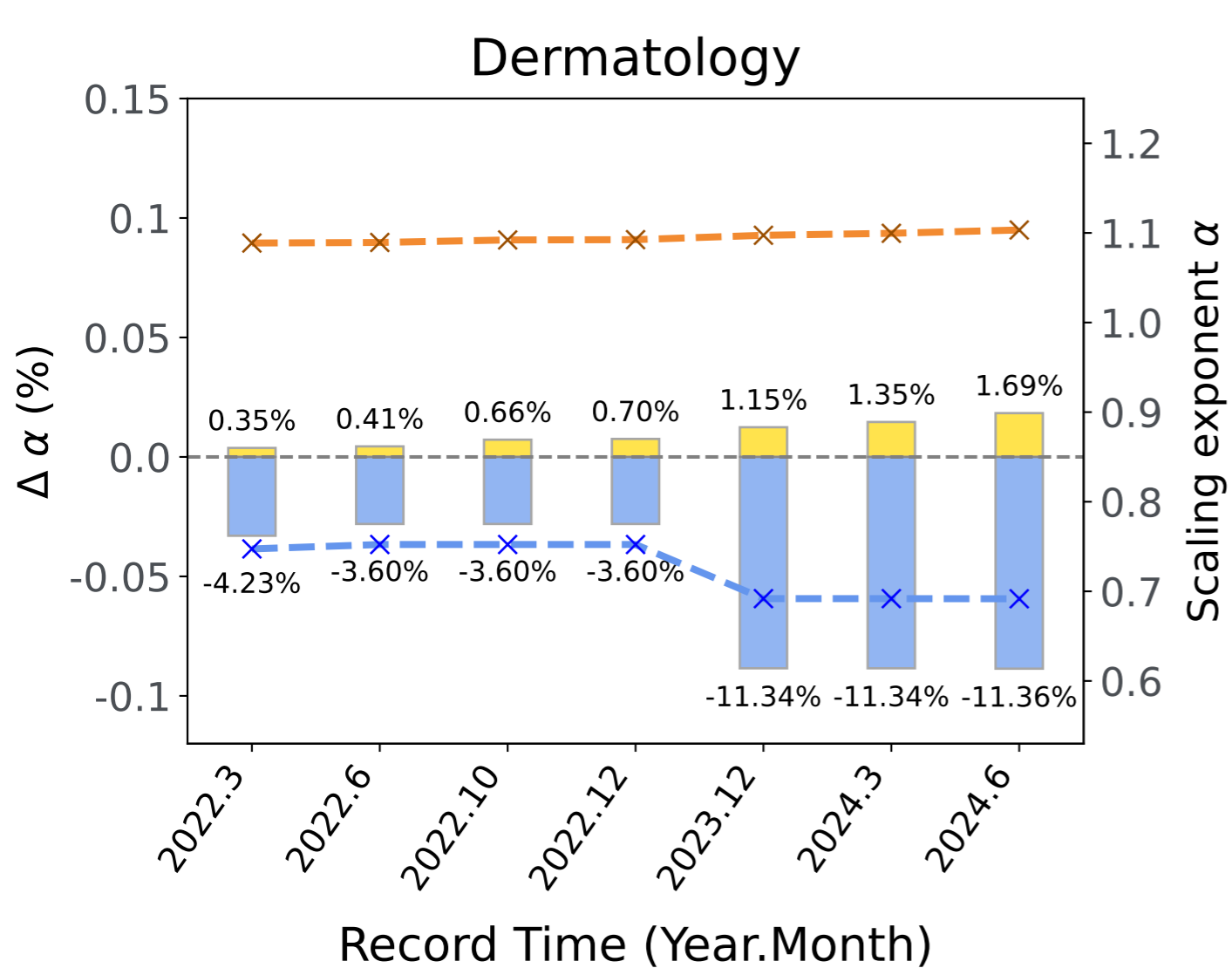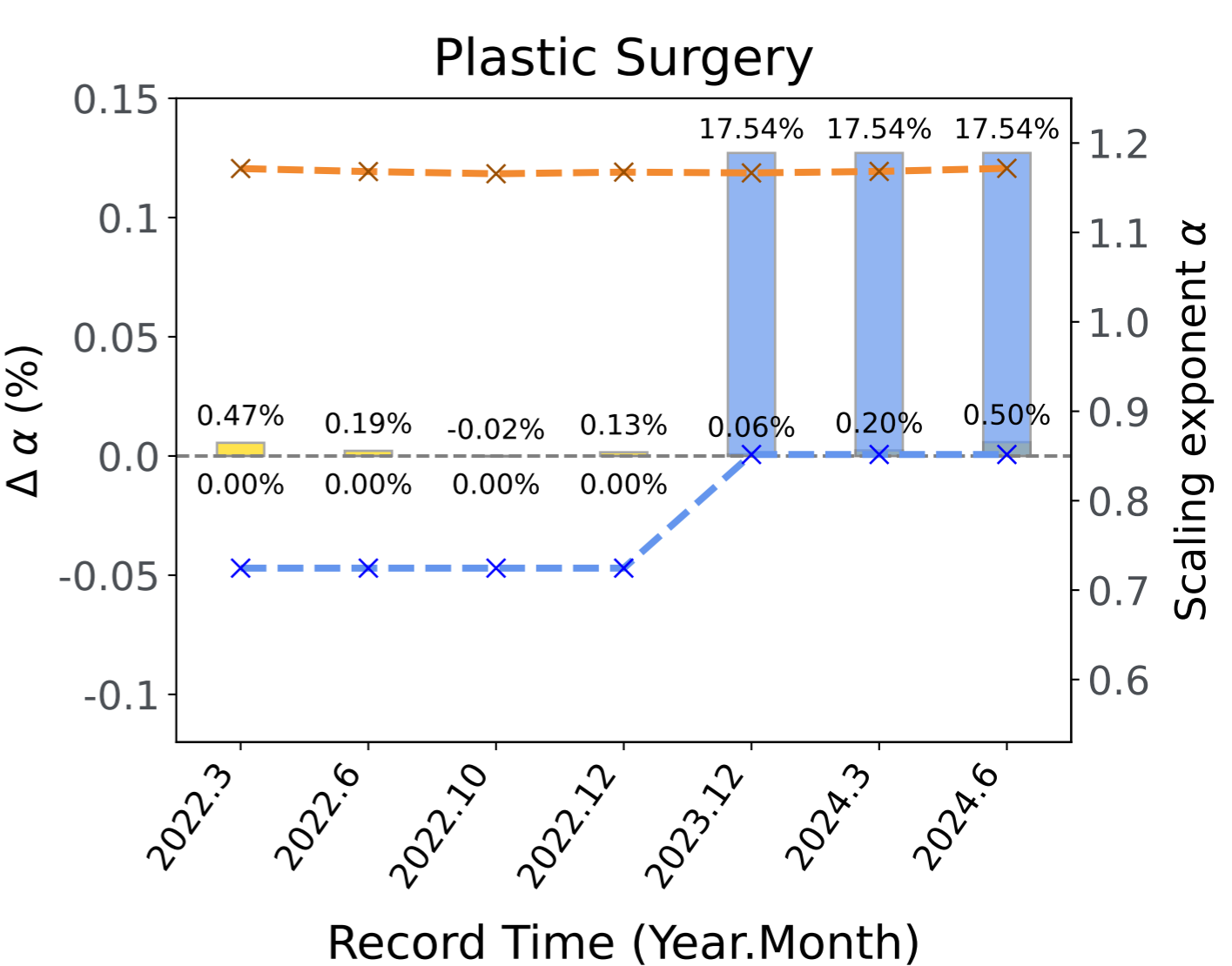

Supplement: S5 Fig — (PDF) [file pone.0330090.s005.pdf]
